# Optimization of the Mechanical and Structural Performance of Bamboo by Microwave–Compression as a Function of Moisture Content

**DOI:** 10.3390/ma18112551

**Published:** 2025-05-29

**Authors:** Huixiang Wang, Rong Liu, Rui An, Xinyu Liu, Shiyu Zhao, Zhaolong Zhu

**Affiliations:** 1 Department of Biological Sciences, Xinzhou Normal University, Xinzhou 034000, China; 2 College of Furnishings and Industrial Design, Nanjing Forestry University, Nanjing 210037, China

**Keywords:** microwave, moisture content, mechanical properties, cell structure

## Abstract

Bamboo, a renewable resource, has broad applications in construction, furniture, and other sectors. However, its dimensional stability and mechanical properties under varying humidity conditions pose challenges. This study aims to investigate the effects of microwave–compression treatment on the mechanical properties, water resistance, and chemical composition of bamboo at various moisture contents, and to elucidate the mechanisms underlying these changes. In the experiment, bamboo samples with moisture contents of 10%, 30%, and 50% were subjected to microwave–compression, and their mechanical properties, water resistance, chemical composition, and microstructure were subsequently analyzed. The results indicate that bamboo with low moisture content (10%) exhibited the best modulus of elasticity (MOE) and modulus of rupture (MOR), while bamboo with higher moisture contents (30% and 50%) showed significant declines in mechanical properties, although dimensional stability improved. Chemical analysis revealed that microwave–compression treatment resulted in the reorganization of lignin and hemicellulose, enhancing the chemical stability of bamboo, while X-ray diffraction (XRD) analysis indicated an increase in crystallinity at higher moisture contents. Overall, the study demonstrates that microwave–compression treatment can optimize the mechanical properties and dimensional stability of bamboo, particularly with moderate moisture contents. The results show that microwave–compression treatment can improve the structural performance of bamboo, especially under low-humidity conditions.

## 1. Introduction

Bamboo—a fast-growing herbaceous grass that thrives across tropical and subtropical Asia, Africa and Latin America—offers a renewable, biodegradable resource with a porous, layered fiber–vessel–parenchyma microstructure that yields an exceptional strength-to-weight ratio [1]. Its short rotation period, high hardness, and toughness make bamboo a practical substitute for increasingly scarce high-quality timber, easing the widening supply–demand gap caused by logging restrictions, dwindling forest resources and rising demand for premium wood products [2]. Currently, bamboo accounts for only 1–3% of the global market in traditional bulk industries such as furniture, flooring, and pulp and paper (approximately 2% for bamboo furniture and 1% for bamboo flooring). However, the bamboo industrial products sector has already reached USD 27.3 billion, making it the largest segment of the entire bamboo industry. Driven by low-carbon regulations and green consumption, bamboo’s compound annual growth rate is generally 2–3 times higher than the corresponding industry average, and it is regarded as one of the most promising sustainable alternative materials [3].

Bamboo is highly renewable and possesses excellent mechanical properties, but it is prone to cracking and exhibits moderate durability. However, bamboo is relatively sensitive to humidity, and high-moisture environments can cause it to absorb moisture and deform, resulting in poor dimensional stability. Additionally, bamboo’s natural growth characteristics may lead to irregular shapes and sizes [3,4]. To enhance bamboo’s performance, it must undergo various treatments such as steaming, compression, and bending [5,6,7,8]. In recent years, the widespread application of microwave technology in material processing has led to the increasing exploration of its potential for modifying bamboo. Compared to traditional heating methods, microwave heating offers advantages such as rapid heating and high energy conversion efficiency, enabling uniform heating of the bamboo interior in a short period for modification. Microwave–compression treatment, an innovative method for modifying bamboo, not only improves its physical and chemical properties, but also holds promise for enhancing its application value.

In Gašparík’s study, samples of beechwood were subjected to microwave heating at 500–700 W, reaching the ideal plasticizing temperature within 2 to 4 min, and when the treatment time reached 6 min, the temperature rose above 100 °C [9]. Furthermore, using microwave technology for pre-treating bamboo showed significant improvements in fixing compressed bamboo. Experiments showed that the swelling rate and deformation recovery rate of compressed wood treated with microwaves were lower than those of other methods, effectively improving the dimensional stability of the compressed material. This improvement is mainly due to the microwaves causing water molecules and polar groups inside the wood to oscillate and convert into thermal energy, resulting in more uniform heating and more even structural deformation and density distribution during compression, thereby reducing the material’s deformation recovery rate.

Despite the increasing use of microwave or compression treatments in lignocellulosic materials, there is still little information on how the combined microwave–compression process affects bamboo across different initial moisture levels. Existing studies usually investigate a single moisture condition or a single treatment, leaving the moisture-dependent synergy between microwaves and mechanical compression largely unexplored.

To address this gap, the present work couples three practical moisture contents (10%, 30%, 50%) with a single-step microwave–compression schedule, quantifies the resulting changes in density, MOE and MOR, and links these macroscale gains to microstructural and chemical evolution via SEM and FT-IR analyses. The study is expected to offer scientific guidance for the bending, compression, and reconstitution of bamboo, while supplying data to optimize processing strategies.

## 2. Materials and Methods

### 2.1. Materials

The bamboo used in this experiment was *Phyllostachys heterocycla* cv. pubescens, sourced from Shaowu City, Fujian Province, and aged 5–6 years. Samples were selected from the middle section of the bamboo (4–6 m above the ground), with the green and yellow portions removed. The bamboo was then processed into strips with dimensions of 100 mm (length) × 20 mm (width) × 8 mm (thickness), and the weight of each sample was recorded.

### 2.2. Microwave-Compression Treatment Method

Bamboo specimens (*n* = 6 per moisture group) were immersed in a covered water bath (25 ± 1 °C, 100% RH) and weighed every 30 min. When the mass increase corresponded to moisture contents (MC) of 10 ± 0.5%, 30 ± 0.5% and 50 ± 0.5% (calibrated by the oven-dry method), the samples were removed, surface water was blotted off with lint-free tissue, and the next step was initiated immediately. The specimens were then exposed to continuous microwave irradiation at 900 W for 120 s in a commercial microwave oven (Galanz G80F22, 2450 MHz, Galanz Electric Co., Ltd., Shunde, Foshan, China). Each sample was placed horizontally on a rotating glass tray to ensure uniform heating; upon completion, the core surface temperature rose rapidly to 110–120 °C, as measured with an infrared thermometer (Testo 830-T2, Testo SE & Co. KGaA, Titisee-Neustadt, Baden-Württemberg, Germany). The hot samples were promptly transferred into a pre-heated steel mold (160 ± 2 °C; inner cavity 300 mm × 20 mm × 10 mm) and positioned between two polished stainless-steel plates of a hot press (MTS Landmark Model 370.25, MTS Systems Corporation, Eden Prairie, MN, USA). Within 15 s of contact, pressure was linearly increased to 5 GPa. Thickness gauges of 2.4 mm were mounted on both sides of the mold to guarantee uniform compression, yielding a final compression ratio of 30%. With pressure maintained, the heating system was switched off; when the press indicated that the temperature had fallen to 60 ± 3 °C (≈2 h), pressure was released linearly to 0 MPa over 60 s, and the compressed bamboo board was removed. Finally, the board was conditioned at 20 °C and 65% relative humidity for 24 h before subsequent mechanical testing and water absorption/thickness swelling evaluations.

### 2.3. Mechanical Property Testing

A three-point bending test was conducted using the Servohydraulic Test System MTS Landmark, as specified in ASTM D7264-15 [10]. Six bamboo strips were selected for the test, with a load of 1 kN, a span of 60 mm, and a displacement control testing speed of 3 mm/min. The bamboo material (100 × 20 × t mm) (length × width × thickness) was oriented with the green side facing the stressed side. The bending strength (MOR) and elastic modulus (MOE) were calculated using the following formulas:

(1) $$\mathrm{MOR}\;\left(\mathrm{MPa}\right)=\frac{3F_{\max}L}{2bd^{2}}$$

(2) $$\mathrm{MOE}\;\left(\mathrm{MPa}\right)=\frac{F_{e}L^{3}}{4\delta bd^{3}}$$

In the above formulas, *F_max_* represents the maximum load (N), *L* is the test span (mm), b is the sample width, and d is the sample thickness (mm). *F_e_* represents the difference in load between the upper and lower boundaries within the proportional limit (N), and *δ* is the deflection at the midpoint of the sample under the load *F_e_* (mm).

### 2.4. Water Resistance Performance Testing

Water absorption (WA) and thickness swelling (TS) tests were conducted following the ASTM D1037–12 standard [11], before and after the microwave–compression treatment of the samples. First, samples measuring 20 × 20 × t mm (length × width × thickness) (6 samples) were dried at 103 ± 2 °C, and their weight and volume were measured. The samples were then fully immersed in water at 22 ± 1 °C. After 12, 24, 36, and 48 h of immersion, the weight and thickness changes were measured. The water absorption rate and thickness swelling rate were calculated using the following formulas:

(3) $$\mathrm{WA}=\frac{m_{t}-{\;m}_{0}}{m_{0}}\;\times \;100\%$$

(4) $$\mathrm{TS}=\frac{t_{t}-t_{0}}{t_{0}}\times 100\%$$

Here, m_0_ represents the dry weight, m_t_ represents the weight of the sample after being immersed in water for 12, 24, 36, and 48 h, t_0_ represents the thickness of the sample after microwave–compression treatment, and t_t_ represents the thickness of the sample after water absorption testing.

### 2.5. Microscopic Structure Testing

The cross-sections of bamboo samples were sliced and dried using a sliding microtome. The surfaces of the samples were then vacuum-coated with platinum for 60 s using an ion sputter (JEOL JFC-1600, JEOL Ltd., Tokyo, Japan) at 30 mA. Scanning electron microscopy (SEM) was conducted using a HITACHI TM3030 (Hitachi High-Tech Corporation, Tokyo, Japan), with a maximum accelerating voltage of 10 kV and a maximum spatial resolution of 2 nm.

### 2.6. Fourier Transform Infrared (FTIR) Testing

FTIR analysis was performed on the samples before and after microwave–compression treatment. A Thermo Scientific (Atomicinstrumen FTIR-650, Nanbei Instrument, Shenzhen, Guangdong, China) spectrometer was utilized for the scan. The sample powder and KBr (Merck 99.99%, Merck KGaA, Darmstadt, Germany) were uniformly ground at a 1:100 ratio. Subsequently, the mixture was compressed for one minute under a pressure of 30 GPa using a tablet press, and it was immediately placed in the spectrometer for scanning to prevent moisture absorption by KBr. The scanning range was from 500 to 4000 cm^−1^ with a resolution of 1 cm^−1^, and the scan was repeated 64 times.

### 2.7. X-Ray Diffraction (XRD) Testing

XRD analysis of the samples was conducted using a Bruker XRD system (Bruker Corporation, Billerica, MA, USA). The X-ray source operated at 30 kV and 20 mA. Scattering radiation was detected in the 2θ range of 5° to 60° at a scan rate of 2°/min. The relative crystallinity (CrI) was determined using the peak area method:

(5) $$\mathrm{CrI}=\frac{A_{1–10}+{\;A}_{110\;}+{\;A}_{012}+{\;A}_{200\;}+{\;A}_{004}}{A_{1–10}+{\;A}_{110}+{\;A}_{012}+{\;A}_{200}+{\;A}_{004}+{\;A}_{\mathrm{am}}}\;\times \;100\%$$

In Equation (5), A_hkl_ represents the peak area of the crystal diffraction peaks, denoted by the Miller indices (hkl). The relative crystallinity (CrI) is calculated based on the areas A_hkl_ of the diffraction peaks (1–10), (110), (012), (200), and (004), which correspond to the crystalline and amorphous regions of the bamboo sample. The total area of the amorphous region is denoted as A_am_.

## 3. Results and Discussion

### 3.1. Mechanical Properties

**Figure 1:**
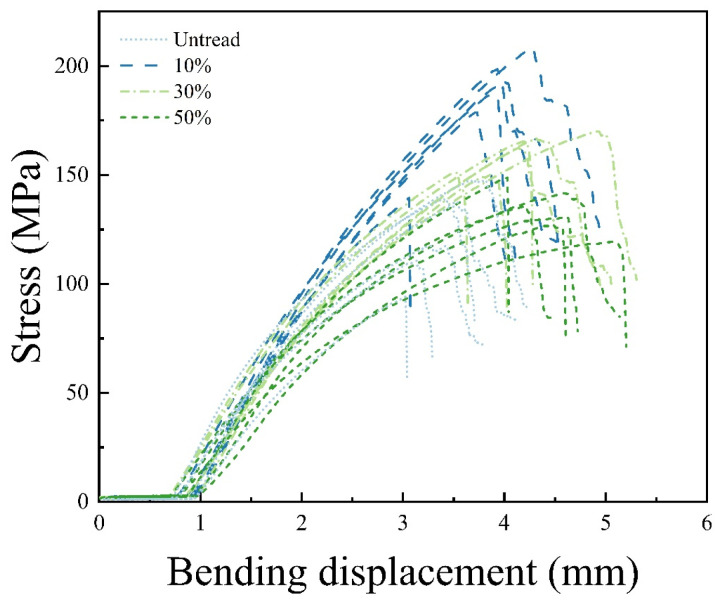
Bending stress–strain curves of bamboo samples treated with microwave–compression and untreated samples.

Figure 1 illustrates the stress–strain curves of untreated bamboo (control group) and bamboo treated with microwave-compression. The control group served as a baseline, with its stress–displacement curve exhibiting fracture at a peak stress of approximately 120 MPa, indicating the limited elastic and plastic deformation capacity of the raw material. For the microwave–compression-treated bamboo, as moisture content increased (10%, 30%, 50%), the corresponding peak stress in the stress–strain curves gradually decreased, while remaining higher than that of the control group. This trend suggests that microwave–compression treatment significantly affects the bamboo’s structure.

From the control group to the 10% moisture content microwave–compression-treated bamboo, the peak stress in the stress–displacement curves gradually increased, indicating that microwave–compression treatment enhanced the material’s load-bearing capacity. As the moisture content increased to 30% and 50%, the peak stress gradually decreased, accompanied by a reduction in bending strength. Among the three moisture content groups, the sample with 50% moisture content exhibited the lowest peak stress.

**Figure 2:**
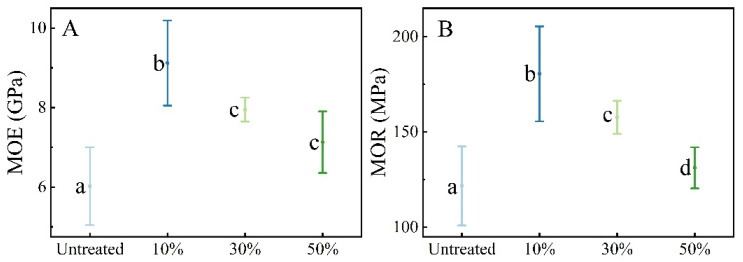
Flexural properties of bamboo samples treated with microwave–compression and untreated samples. (**A**) Bending strength (MOR). (**B**) Modulus of elasticity (MOE). Different lowercase letters (a, b, c, d) indicate statistically significant differences among groups (*p* < 0.05) according to one-way ANOVA followed by Tukey’s post-hoc test.

Figure 2A,B show that initial moisture content significantly impacts the elastic modulus and bending strength of treated bamboo. The average MOE and MOR of the control group were 6.02 ± 0.931 GPa and 121.68 ± 19.792 MPa, respectively. Bamboo with 10% moisture content, following microwave–compression treatment, exhibited the highest elastic modulus and bending strength, at 9.12 ± 1.020 GPa and 180.60 ± 23.724 MPa, respectively. Bamboo flooring compressed at a 50% ratio typically achieves an MOR increase of over 70% and an MOE increase of about 30% compared to untreated bamboo [12]. This is likely because, at this moisture content, the bamboo’s fiber bundle structure maintains relatively high strength and is more compact, resulting in superior stiffness and strength after compression. At 30% moisture content, the fiber bundle structure became more dispersed after microwave treatment, resulting in a reduction in the overall strength of the bamboo. Related work showed that during the microwave treatment of bamboo that the surfaces of the fiber bundles developed “sheet-like” cracks, the parenchyma cells contracted, and the interface between the bundles and the surrounding matrix became loosened [13]. Bamboo with 50% moisture content, due to the large volume of water evaporating, experienced extremely high steam pressure and thermal stress, leading to severe structural degradation and the poorest mechanical properties. This corresponds to the findings of Wang Y’s experiments, which noted that when the moisture content is excessively high, microwave treatment causes the internal steam pressure within the bamboo to rise rapidly in a short time, readily triggering cell wall buckling and cracking [14].

In conclusion, initial moisture content plays a key role in optimizing the plasticization process of bamboo. An optimal initial moisture content can enhance the material’s strength and stability. During microwave processing, the rapid heating of water within the bamboo leads to lignin softening [15]. During subsequent compression, the fiber strands are more easily rearranged, and the cell cavities are more readily compressed, thereby increasing the material’s density. This microscopic reorganization leads to improved macroscopic bending performance [16]. However, an excessively high moisture content generates extremely high internal steam pressure and thermal stress during evaporation, severely damaging the cell structure. Excessive degradation and reorganization result in changes in the lignin and hemicellulose structure, forming unstable chemical structures, which reduce the material’s mechanical properties [17].

### 3.2. Water Absorption and Dimensional Stability

**Figure 3:**
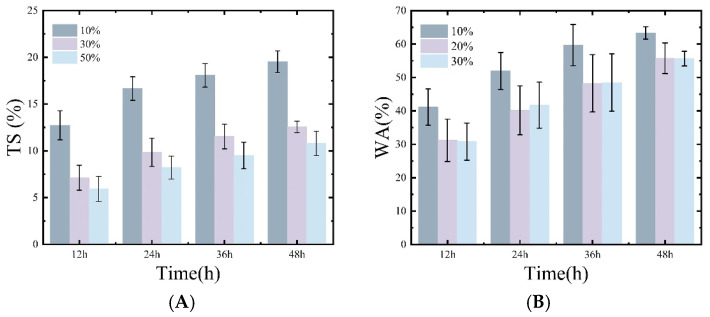
The water absorption rate and thickness swelling rate of microwave–compression-treated and untreated samples after soaking for 12 h, 24 h, 36 h, and 48 h are shown in (**A**) water absorption rate and (**B**) thickness swelling rate.

Figure 3 illustrates the water absorption rate of bamboo treated with microwaves at different powers over 12, 24, 36, and 48 h. The water absorption rate increased over time, particularly between 0 and 12 h. This phenomenon suggests that the water absorption process is more active in the early stages and gradually levels off thereafter. As shown in the chart, at all time points, bamboo treated with 10% moisture content had the highest water absorption rate, indicating that bamboo at this moisture content absorbed the most water (48 h—63.330%). This could be due to the moderate porosity developed within the bamboo treated with 10% moisture content, which allows it to absorb more water without blocking the pores, thereby retaining its water absorption capability. Lv et al. also observed similar results in his experiments, finding that the samples’ specific surface area and pore volume were directly proportional to their water adsorption capacity [14]. In contrast, the water absorption rates of the 30% and 50% moisture content groups were relatively lower (at 48 h, they were 55.772% and 55.623%, respectively), likely due to the microwave treatment increasing the internal structural density while maintaining the integrity of the fiber bundles, which hindered water absorption [18].

Similarly, bamboo with 10% moisture content exhibited the highest thickness swelling rate, as the well-preserved fiber bundle structure allowed the bamboo to better recover its original form after absorbing water, thereby reducing its dimensional stability [19]. In the 30% and 50% moisture content groups, the thickness swelling rates were lower, suggesting that microwave treatment at higher moisture contents facilitated the crosslinking of cellulose, hemicellulose, and lignin, resulting in a more stable structure. This stable structure is less prone to significant swelling during water absorption, thus maintaining better dimensional stability [20,21,22]. The dimensional stability achieved by bamboo compressed under microwave treatment is far superior to that of bamboo processed by conventional hot pressing. For example, in Kadivar’s experiments, bamboo subjected to conventional hot pressing exhibited a thickness recovery rate of 17.7% [23].

### 3.3. FTIR Analysis

**Figure 4:**
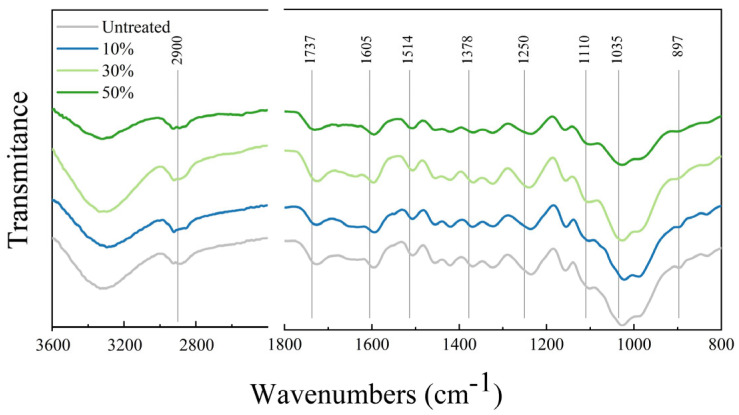
FTIR spectra of bamboo samples treated with microwave–compression and untreated samples.

To investigate the impacts of varying moisture contents on bamboo during the microwave–compression treatment process, chemical composition analyses were conducted on both the control group and the microwave–compression-treated bamboo. Figure 4 presents the FTIR spectra of the control group and the microwave–compression-treated bamboo samples in the range of 800–3700 cm^−1^. Considering that the C-H aliphatic stretching vibration (around 2900 cm^−1^) is more stable during thermal treatment than C-OH, C-O-C, and R-COO-R [24,25], the peak intensity ratios were calculated by comparing the intensities of the peaks at 1737, 1605, 1514, 1378, 1250, 1110, 1035, and 897 cm^−1^ with the peak at 2900 cm^−1^, as presented in Table 1.

I_1737_/I_2900_, I_1605_/I_2900_, I_1514_/I_2900_, I_1375_/I_2900_, I_1378_/I_2900_, I_1250_/I_2900_, I_1110_/I_2900_, I_1035_/I_2900_, and I_896_/I_2900_, represent the relative peak intensity ratios of each peak to the peak at 2900 cm^−1^. These ratios correspond to the intensity of the peaks at 1737, 1605, 1514, 1378, 1250, 1110, 1035, and 897 cm^−1^ relative to the intensity of the 2900 cm^−1^ peak.

The peak at 1737 cm^−1^ corresponds to the stretching vibration of the carbonyl group (C=O) in hemicellulose, which decreases as moisture content increases. However, when the moisture content reaches 50%, this peak intensity increases. This suggests that, at a moderate moisture content, microwave treatment induces the partial degradation of the carbonyl groups in hemicellulose, whereas at higher moisture contents, it may facilitate the reorganization of hemicellulose, forming more carbonyl structures [17]. The changes in peak intensity at 1605 cm^−1^, 1369 cm^−1^, 1250 cm^−1^, and 896 cm^−1^ exhibit a similar trend, with peak intensity first decreasing and then increasing as moisture content rises. This indicates that, at a moderate moisture content, microwave treatment may cause the partial degradation of lignin [26]. Lv et al. also observed that microwave radiation induces the cleavage of aromatic lignin rings (C=C) in the bamboo cell wall structure [13]. Additionally, at a higher moisture content, microwave treatment reduces the degradation of hemicellulose and promotes the esterification of lignin, forming new lignin-related alcohol and ester compounds. These chemical changes increase the infrared absorption peak intensities at specific wavelengths, such as those corresponding to the C=O and C–H vibrations [17].

At moderate and low moisture contents, water exerts a plasticizing effect, but also leads to the considerable degradation of lignin and hemicellulose. The energy from microwave treatment is partially utilized for moisture evaporation and partially for heating, resulting in chemical structure degradation and a reduction in absorption peak intensities [27]. At a high moisture content, water functions as a plasticizer, and microwave treatment induces the thermal reorganization of lignin and hemicellulose at elevated temperatures, forming new chemical bonds and structures, which results in an increase in absorption peak intensities [28,29].

### 3.4. Changes in Crystallinity

Cellulose plays a key role in supporting the structure of bamboo. The relationship between its amorphous and crystalline components influences the mechanical properties and hydrophilicity of bamboo [30]. When bamboo is heated, moisture accelerates the pyrolysis of cellulose and hemicellulose [31]. Therefore, XRD analysis was performed on both the control group and the microwave–compression-treated bamboo to observe changes in relative crystallinity.

**Figure 5:**
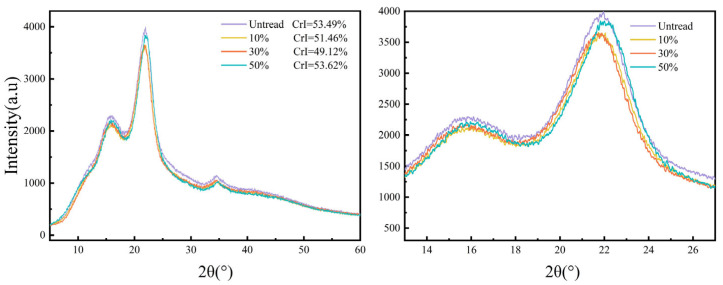
XRD patterns of bamboo samples treated with microwave–compression and untreated samples.

Figure 5 presents the X-ray diffraction (XRD) patterns of the control group and bamboo treated with microwaves at varying moisture contents. The control group exhibited a crystallinity of 53.49%, indicating a high proportion of crystalline regions in the cellulose and a stable structure. The 10% moisture content group exhibited a crystallinity of 51.46%, with microwave treatment causing the slight degradation of some crystalline regions in cellulose; however, the impact was minor, and the material maintained a high degree of crystallinity. The 30% moisture content group exhibited a crystallinity of 49.12%, lower than that of the 10% moisture content group. At this moisture level, microwave treatment caused the further degradation of cellulose crystalline regions and the partial reorganization of hemicellulose, leading to the formation of amorphous regions [32]. The 50% moisture content group exhibited the highest crystallinity at 53.62%. At high moisture content, microwave treatment resulted in the thermal reorganization and partial degradation of lignin and hemicellulose. These chemical changes reduced the proportion of amorphous regions and increased the relative proportion of crystalline regions [13]. Additionally, under high temperature and high steam pressure, the cellulose molecular chains may rearrange, forming more crystalline regions, which, in turn, increases the overall crystallinity.

### 3.5. Changes in Cell Structure

**Figure 6:**
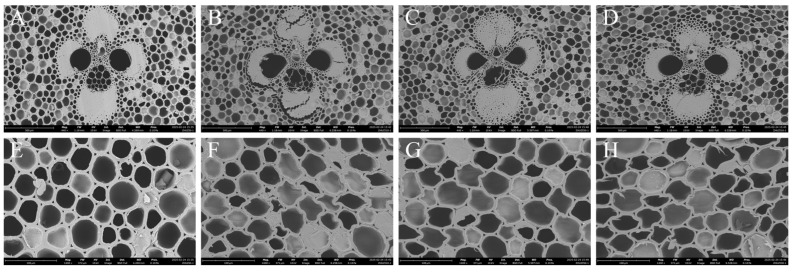
(**A**,**E**) Electron micrographs of the control group bamboo. (**B**,**F**) Electron micrographs of bamboo treated with microwave–compression at 10% moisture content. (**C**,**G**) Electron micrographs of bamboo treated with microwave–compression at 30% moisture content. (**D**,**H**) Electron micrographs of bamboo treated with microwave–compression at 50% moisture content.

In Figure 6, Figure 6A,E display the original structure of the control group bamboo, characterized by a relatively uniform and well-defined pore structure. For bamboo with 10% moisture content, Figure 6B,F illustrate significant deformation of the fiber bundle structure, accompanied by localized rupture. The deformation of the parenchyma cells is uneven, with some areas undergoing significant deformation while others remain almost unaffected. This phenomenon could be attributed to the rapid evaporation of moisture during microwave–compression in bamboo at lower moisture contents, leading to insufficient softening and resulting in localized stress variations. These variations then result in cell wall rupture or significant deformation during the hot press process. Despite the rupture of some cell walls, bamboo at a 10% moisture content exhibited higher elastic modulus (MOE) and bending strength (MOR), suggesting that the fiber bundle structure of bamboo maintains high strength in lower moisture conditions with a more compact structure. This imparts better rigidity and strength to the bamboo, although it may also allow the bamboo to recover its original structure more effectively when absorbing water, potentially lowering its dimensional stability [33,34].

For bamboo with a 30% moisture content, Figure 6C,G show that the fiber bundles undergo deformation without rupture, with the deformation being more uniform compared to the 10% moisture content samples. The deformation of the parenchyma cells is more uniform, with no significant localized rupture observed. At 50% moisture content, the bamboo fiber bundles undergo significant deformation, and the deformation of the cell walls becomes more pronounced. This indicates that, under moderate- and high-moisture conditions, the bamboo structure deforms more stably, with cell walls being relatively soft and less prone to rupture.

In terms of mechanical properties, bamboo with 30% and 50% moisture contents exhibited lower elastic modulus (MOE) and moderate bending strength (MOR) compared to bamboo with 10% moisture content. This suggests that the fiber bundle structure becomes more loosened after microwave treatment at higher moisture contents, leading to a decrease in overall bamboo strength. Although the deformation is more uniform, the overall strength is reduced. While XRD analysis shows a slight increase in crystallinity, the severe degradation of the chemical structure significantly reduces the overall mechanical properties and internal stress of the bamboo, while also improving dimensional stability [34].

## 4. Conclusions

In summary, this study proposes a microwave pretreatment plus compression modification method for bamboo, which markedly enhances the mechanical performance of the material. Compared with untreated bamboo, the longitudinal flexural modules of elasticity (MOE) of the treated specimens increased by roughly 30%, the modulus of rupture (MOR) rose by about 25%, and density also increased. This innovative approach uses microwave heating to rapidly soften bamboo cell walls, allowing the material to be compressed and set at lower temperatures and in a shorter time, thereby demonstrating high efficiency and practicality. Without the use of chemical reagents, it can effectively strengthen bamboo, improve material utilization, and broaden its application potential in engineering fields.

Despite these achievements, the study has certain limitations. First, microwave treatment may cause localized micro-cracks or component degradation in bamboo cell structures. Second, although microwave–compressed bamboo shows better dimensional stability, its water absorption rate remains high, with post-soaking mass still exceeding half of its own dry weight. This indicates that the material’s durability in humid environments is still inadequate. Therefore, practical applications will require subsequent hydrophobic treatments or further optimization of the microwave–compression process to reduce water absorption, along with long-term performance evaluations of microwave-treated bamboo. This will also be a primary focus in the next phase of our research.

## Figures and Tables

**Table 1:** Relative peak intensity ratios of bamboo treated with microwave–compression and untreated bamboo.

	I_1737_/I_2900_	I_1605_/I_2900_	I_1514_/I_2900_	I_1378_/I_2900_	I_1250_/I_2900_	I_1110_/I_2900_	I_1035_/I_2900_	I_896_/I_2900_
Untreated	0.8858	0.9058	0.8575	0.9027	0.9080	0.9731	1.1282	0.9098
10%	0.8777	0.8864	0.8777	0.8864	0.8777	0.8864	0.8777	0.8864
30%	0.8312	0.8486	0.8090	0.8476	0.8484	0.9241	1.1332	0.8384
50%	0.9484	0.9731	0.9575	0.9659	0.9524	1.0088	1.0959	0.9649

## Data Availability

The original contributions presented in this study are included in the article. Further inquiries can be directed to the corresponding author.
